# Enhanced Performance of AlGaN-Based Deep Ultraviolet Light-Emitting Diodes with Chirped Superlattice Electron Deceleration Layer

**DOI:** 10.1186/s11671-019-3201-x

**Published:** 2019-11-21

**Authors:** Jiahui Hu, Jun Zhang, Yi Zhang, Huixue Zhang, Hanling Long, Qian Chen, Maocheng Shan, Shida Du, Jiangnan Dai, Changqing Chen

**Affiliations:** Wuhan National Laboratory for Optoelectronics, Huazhong University of Science and Technology, Wuhan, 430074 China

**Keywords:** AlGaN, DUV LED, SEDL, MOCVD, APSYS

## Abstract

AlGaN-based deep ultraviolet (DUV) light-emitting diodes (LEDs) suffer from electron overflow and insufficient hole injection. In this paper, novel DUV LED structures with superlattice electron deceleration layer (SEDL) is proposed to decelerate the electrons injected to the active region and improve radiative recombination. The effects of several chirped SEDLs on the performance of DUV LEDs have been studied experimentally and numerically. The DUV LEDs have been grown by metal-organic chemical vapor deposition (MOCVD) and fabricated into 762 × 762 μm^2^ chips, exhibiting single peak emission at 275 nm. The external quantum efficiency of 3.43% and operating voltage of 6.4 V are measured at a forward current of 40 mA, indicating that the wall-plug efficiency is 2.41% of the DUV LEDs with ascending Al-content chirped SEDL. The mechanism responsible for this improvement is investigated by theoretical simulations. The lifetime of the DUV LED with ascending Al-content chirped SEDL is measured to be over 10,000 h at L50, due to the carrier injection promotion.

## Introduction

In recent years, AlGaN-based deep ultraviolet (DUV) light-emitting diodes (LEDs), whose spectra ascribed to UVB (320 nm–280 nm) and UVC (280 nm–100 nm), have attracted much attention because of their applications in plant lighting, phototherapy, water purification, and air and surface sterilization [1–6]. However, the light output power (LOP) of the state-of-the-art AlGaN-based DUV LEDs drops significantly as the light emission wavelength gets shorter [7, 8]. Those DUV LEDs suffer from low internal quantum efficiency (IQE), light extraction efficiency (LEE), and carrier injection efficiency (CIE) [9–13]. Generally, deficient IQE is caused by large density of defects and threading dislocations, while insufficient LEE is due to the polarization of AlGaN materials and the absorption by the nontransparent p-GaN contact layer [14–18]. Furthermore, electron overflow is the main reason for the poor CIE, which is on account of the inadequate hole density and the significantly imbalanced mobility of electron and hole in AlGaN materials [19, 20].

Conventionally, high-Al-content p-type AlGaN electron blocking layer (EBL) is used to suppress the electron overflow. But only a few holes can be injected into the active region through the barrier in the valence band introduced by the EBL, and even less holes can cross the barriers of the active region and transport to the quantum wells near n-type layers because of low activation efficiency of the Mg dopant and small mobility of holes [21]. Various attempts have been made to improve electron and hole injection, such as hole barrier layer, specifically designed last barrier, EBL, and multiple quantum well structures [22–26]. Nevertheless, the performance of DUV LEDs is not substantially improved.

In this work, we have proposed a novel DUV LED structure with superlattice electron deceleration layer (SEDL) to decelerate the electron injection and restrain the electron overflow without compromising the hole injection. We have studied the effects of several SEDLs on the performance of DUV LEDs experimentally and numerically. The DUV LEDs have been grown by metal-organic chemical vapor deposition (MOCVD) and fabricated into 762 × 762 μm^2^ chips, exhibiting single peak emission at 275 nm. The external quantum efficiency (EQE) of 3.43% and operating voltage of 6.4 V were measured at a forward current of 40 mA, indicating that the wall-plug efficiency is 2.41% of the DUV LEDs with ascending Al-content chirped SEDL. The lifetime of the DUV LED with ascending Al-content chirped SEDL is measured to be over 10,000 h at L50. Furthermore, the mechanism of performance enhancement is investigated by theoretical simulation. It is verified that chirped SEDLs are able to equilibrate electron and hole injection into the active region, which promotes the radiative recombination in the first few quantum wells near n-type layers.

## Methods and Experimental Section

### Epitaxy by MOCVD

**Fig. 1:**
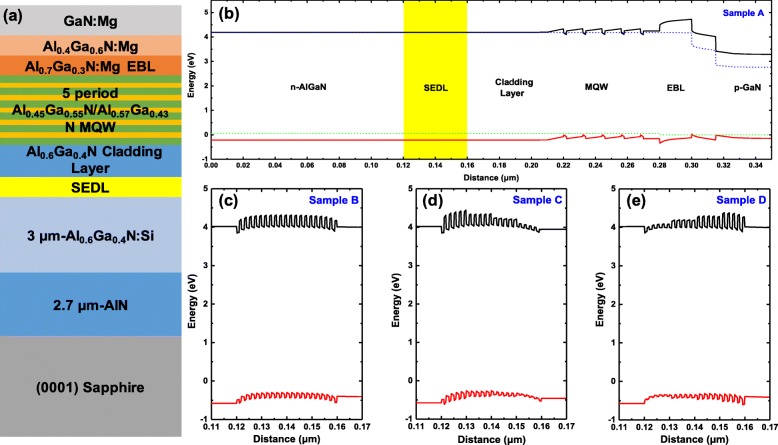
Simulation of the designed structures of DUV LED with and without SEDL. **a** A schematic of DUV LED structure with chirped SEDL. The 20-period SEDL with different Al compositions is inserted between the n-type AlGaN layer and the AlGaN current spreading cladding layer. **b** Whole band structure of conventional sample (**a**) without SEDL. The highlighted area refers to the designate region where the SEDL is to be inserted. **c** Band structure of the SEDL of sample (**b**), which is the 20-period homogeneous Al0.5Ga0.5N/Al0.65Ga0.35N superlattice. Each layer of the SEDL is 1 nm. **d** Band structure of the SEDL of sample (**c**), which is four sets of the 5-period declining Al-content SEDL superlattice with different high-Al-content layers, namely 0.7, 0.65, 0.6, and 0.55. **e** Band structure of the SEDL of sample (**d**), which is four sets of the 5-period ascending Al-content SEDL superlattice with different high-Al-content layers, namely 0.55, 0.6, 0.65, and 0.7

AlGaN-based DUV LED heterostructures were grown using a vertical cold-wall MOCVD system. For the epitaxy of the whole structure, trimethylaluminum (TMA), trimethylgallium (TMG), and ammonia (NH_3_) were used as the Al, Ga, and N sources, respectively. H_2_ was used as the carrier gas. Figure 1a illustrates the schematic for the DUV LED structure with chirped SEDL. The growth was initiated with a 2.7-μm-thick AlN, using the growth method with initial AlN gradient interlayer for growth mode modification [27], then a 3-μm-thick Si-doped Al_0.6_Ga_0.4_N n-type contact layer, of which the electron concentration and mobility of this n-type layer are measured to be 4.5 × 10^18^ cm^−3^ and 52 cm^2^/V s, respectively, by Hall system. It is followed by the 40-nm-thick undoped SEDL. Figure 1b–e. shows the band structures of the conventional DUV LED and three proposed DUV LED with SEDL, named samples A, B, C, and D, respectively. As exhibited in Fig. 1c, sample B has a uniform SEDL of 20-period homogeneous Al_0.65_Ga_0.35_N/Al_0.5_Ga_0.5_N superlattice. The chirped SEDLs of samples C and D are composed of four sets of 5-period superlattice with different high-Al-content layers, namely, 0.7, 0.65, 0.6, and 0.55, while the Al composition of low-Al-content layers is kept constant to be 0.5. For sample C, the Al compositions of high-Al-content layers are gradually rising from bottom to top, which is contrary to that for sample D, as shown in Fig. 1 d and e. The thicknesses of each layer for SEDL are set to be 1 nm steadily. The active region of DUV LEDs consists of an Al_0.6_Ga_0.4_N:Si cladding layer for current spreading, followed by a 5-period multiple quantum wells, using 14-nm-thick Al_0.57_Ga_0.43_N barriers and 2-nm-thick Al_0.45_Ga_0.55_N wells. Then, Al_0.7_Ga_0.3_N:Mg EBL and GaN:Mg p-type contact layer were grown in sequence. The hole concentration and mobility of p-GaN is measured to be 3.6 × 10^17^ cm^−3^ and 15 cm^2^/V s, respectively, by Hall system.

### Device Fabrication

Following the MOCVD growth, DUV LEDs were fabricated with standard processing techniques. First, mesa structures with square and finger geometries were formed by dry-etching down to 150 nm below the top of Si-doped Al_0.6_Ga_0.4_N n-type contact layer, followed by a 900 °C annealing to repair the etching damage. Then, Ti/Al/Ni/Au n-contact metal stack was deposited and annealed at 850 °C in nitrogen atmosphere. Subsequently, an ITO film was evaporated and annealed at 250 °C for the use of p-contact, followed by thick electrode evaporation, passivation layer deposition, pad evaporation, and stealth dicing into 762 × 762 μm^2^ chips.

### Simulation

To illuminate the mechanism of performance enhancement of DUV LEDs, the band diagram, optical properties, and carrier transport characteristics of this structure were simulated by solving the Schrödinger equation, Poisson’s equation, the carrier transport equations, and the current continuity equation self-consistently by Crosslight APSYS (Advance Physical Model of Semiconductor Devices) programs [28]. The Shockley-Read-Hall (SRH) recombination time is set to be 1.5 ns for all layers except the p-type inserted layer as 1 ns because the SRH lifetime is dependent upon the doping level [29]. The internal loss is 2000 m^−1^ [30]. The bowing parameter *b* is 1 eV, and the band-offset ratio is assumed to be 0.7/0.3 for AlGaN materials [31]. The Auger recombination coefficient is set to be 1 × 10^−30^ cm^6^/s to fit the experiment [32]. In this simulation, the built-in interface charges due to the spontaneous and piezoelectric polarization are calculated based on the method proposed by Fiorentini et al. [33]. Furthermore, taking the screening by defects into consideration, the surface charge densities are assumed to be 40% of the calculated values [34].

## Results and Discussion

As four samples possess the identical AlN and n-type AlGaN templates, the crystalline qualities of samples A, B, C, and D were measured by high-resolution X-ray diffraction (HR-XRD). As shown in Table 1, X-ray rocking curves (XRC) along symmetric (002) plane and asymmetric (102) plane for both layers were performed. The results show that the XRC full width at half maximum (FWHM) and threading dislocation density (TDD) of four samples are nearly the same, indicating that the crystalline quality is not the main reason for the device performance improvement. Furthermore, it could be found that threading dislocation densities (TDDs) in the AlGaN layer is higher than those in the AlN layer, which resulted from mixed crystal properties, interface defects, and Si-doping impurities [35]. According to the research of Ban et al. about the correlation between IQE and TDD, the IQE for all samples in this work is approximately 30–40% [36].

**Table 1:** Crystalline quality characterization of AlN and n-type AlGaN layers of samples A, B, C, and D by high-resolution X-ray diffraction along symmetric (002) plane and asymmetric (102) plane. Threading dislocation density (TDD) was calculated according to ref. [27]

Sample	FWHM				TDD	
	AlN-(002) (arcsec)	AlN-(102) (arcsec)	AlGaN-(002) (arcsec)	AlGaN-(102) (arcsec)	AlN (cm^−3^)	AlGaN (cm^−3^)
A	356	345	371	402	8.87 × 10^8^	1.33 × 10^9^
B	352	343	368	397	8.83 × 10^8^	1.30 × 10^9^
C	357	344	374	405	8.76 × 10^8^	1.35 × 10^9^
D	350	339	373	396	8.56 × 10^8^	1.27 × 10^9^

**Fig. 2:**
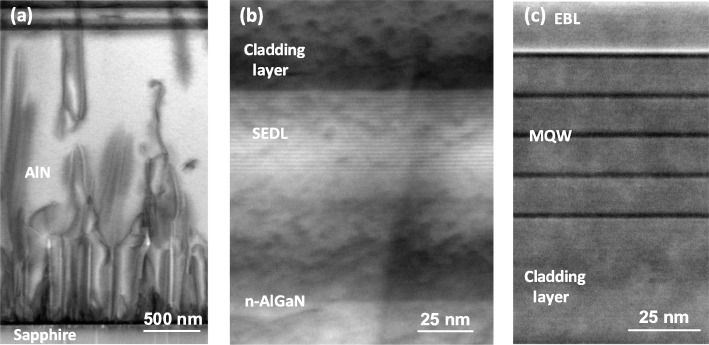
Morphology characterization of typical sample B. **a** Cross-sectional STEM image of the AlN template. **b** Cross-sectional STEM image in the region of 20 periods of SEDL. **c** Cross-sectional STEM image in the active region

To confirm the successful growth of the novel structure, we performed cross-sectional bright-field scanning transmission electron microscopy (BF-STEM) measurements for typical sample B as a representative, as shown in Fig. 2. It can be seen that the TDDs decrease during whole growth process of the 2.7-μm-thick AlN in Fig. 2a. Figure 2b indicates good periodicity and nearly 1-nm-thick layer in each period of SEDL. Furthermore, five periods of multiple quantum wells with distinct interfaces are recognized in Fig. 2c, of which barriers are 14 nm and wells are about 2.1 nm.

**Fig. 3:**
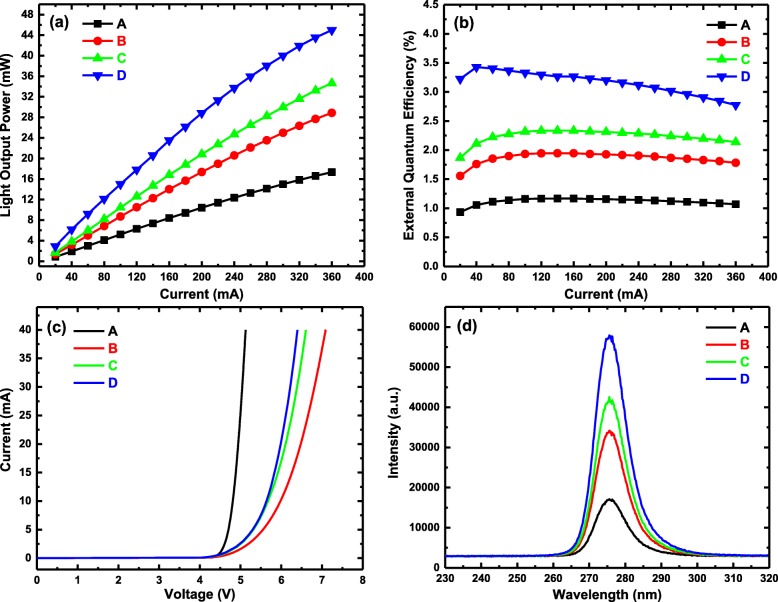
Electrical and optical characteristics of samples with different SEDLs at room temperature. **a** Dependence of LOP on injection current under the CW biases. **b** Dependence of EQE on injection current under the CW biases. **c** Dependence of injection current on operation voltage. **d** EL spectra of all the samples at 10-mA injection current, the peak emissions of which are around 275 nm

In order to investigate the device performance, chips of DUV LED were eutectic bonded on AlN ceramic substrate to minimize the heating effect. Afterwards, the substrate was mounted on a hexagonal aluminum plate by solder paste. Then, electrical and optical measurements were performed, using ATA-1000 Photoelectric Analysis System equipped with a 30-cm-diameter integrating sphere [37]. Figure 3a shows the variations of the light output power (LOP) versus injection current. The LOPs of sample D with ascending Al-content SEDL are 6.17 mW at 40 mA, 14.99 mW at 100 mA, and 44.975 mW at 360 mA, which is a factor of three times higher than that of conventional sample A without SEDL. This indicates that SEDL is beneficial for electron overflow suppression and hole injection. Meanwhile, slight LOP saturation for four samples can be observed, when operating at high biases, which is related to the heating effect and Auger recombination [38]. The EQE against injection current is depicted in Fig. 3b. The maximum EQE is 3.43% at 40 mA for sample D, while the EQE peaks at only 1.17% for sample A. Meanwhile, the LOP and EQE of sample D with ascending Al-content SEDL are higher than those of sample B with uniform and declining Al-content SEDLs, which demonstrates more efficient radiative recombination in sample D. The measured current-voltage characteristics for all the samples are shown in Fig. 3c. It can be recognized that the incorporation of SEDLs increases the operation voltage from 5.13 V at 40 mA for sample A to 7.09 V at 40 mA for sample B, due to the resistivity augment of the high Al composition SEDL. In addition, it can be seen that the operation voltage is lower for samples C and D than for sample B. According to the structure design and the transmission measurement for the single-layer samples, the average Al composition of the barriers of sample C and D SEDL is 62.5% while that of sample B is 65%. The higher Al content leads to lower doping efficiency and higher resistance, resulting to the increase of the operation voltage. It is worth to mention that the voltage of sample D is 6.4 V at 40 mA, resulting in the maximum wall-plug efficiency (WPE) of 2.41%. The electroluminescence spectra at 10 mA are shown in Fig. 3d. The peak emissions of four samples are all around 275 nm, and the trend of peak intensity is the same as LOP. This also indicates that the ascending Al-content chirped SEDL is available for the enhancement of DUV LED device performance.

**Fig. 4:**
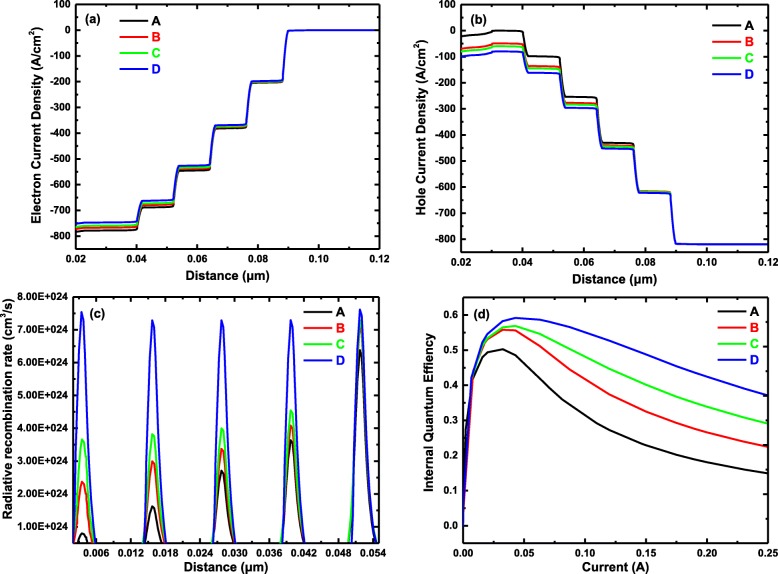
Theoretical simulations and analysis. **a** Electron current density in the active region at the injection current of 200 mA. **b** Hole current density in the active region at the injection current of 200 mA. **c** Radiative recombination rate in the multiple quantum wells at the injection current of 200 mA. **d** Dependence of calculated IQE on the injection current

To shed light on the mechanism responsible for this improvement, theoretical simulations were performed by APSYS program and the results are displayed in Fig. 4. The electron current density and the hole current density distributions near the active region at 200 mA are calculated in Fig. 4 a and b. It could be found that the electron injection current densities of samples with SEDL are slightly lower than those of sample A without SEDL, while the situation is inverse for the hole injection current, illustrating that SEDL is able to decelerate the electron from the n-type AlGaN electron injection layer and promote the hole injection accordingly. The radiative recombination rates for all the samples were calculated in Fig. 4c. With the incorporation of different SEDLs, the radiative recombination rate in the quantum wells near the n-type layer is obviously increased. Meanwhile, from sample A to sample D, the radiative recombination rates in the five quantum wells are gradually becoming uniform, which is almost the same for the sample D with ascending Al-content chirped SEDL. This further indicates that SEDL can equilibrate the injection of electron and hole carriers into the active region and promote the radiative recombination in the first few quantum wells near n-type layers at the meantime. As a result, the IQEs for the four samples were simulated and plotted in Fig. 4d. The IQE of sample D is the highest, which is consistent with the EQE in Fig. 4b. What is more, the efficiency droop in the sample with SEDL is improved apparently. In the whole injection current range, the efficiency droop is 70.33%, 59.79%, 48.93%, and 36.26% for samples A, B, C, and D, respectively, which is defined as the efficiency droop = (IQE_max_ − IQE_250 mA_)/IQE_max_. The efficiency droop is generally thought to be caused by electron leakage and insufficient hole injection [39]. The improvement of efficiency droop clarifies that SEDL can balance the carrier transport to the active region and promote the radiative recombination in the quantum wells, enhancing the device performance ultimately.

**Fig. 5:**
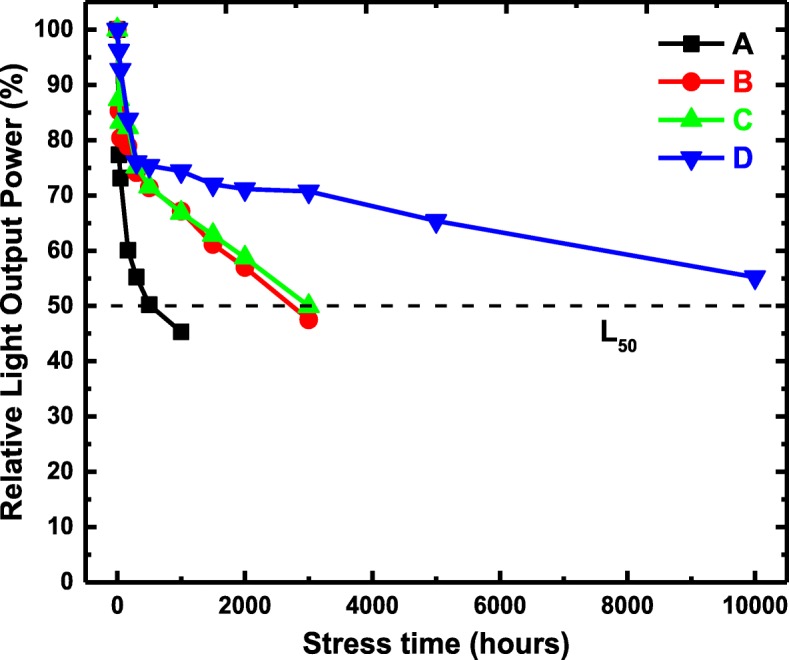
The relative LOP as a function of the aging time for all the samples at 20 mA and room temperature. The aging is stopped when the relative LOP is under 50%. Black, red, green, and blue curves represent samples **a**, **b**, **c**, and **d**, respectively. The lifetime for sample D with ascending Al-content chirped SEDL is over 10,000 h at L50

The lifetime of the devices was measured at 20 mA and room temperature. For each sample, to ensure the accuracy of the results, 10 chips were random selected and the average of the relative LOP of them at different stress time was depicted in Fig. 5. As is shown, compared to sample A, the lifetime of samples with SEDL is obviously extended. The degradation of LED devices is partly related to the defect accumulation, ohmic conductive channels, and deficient carrier injection [40]. The improvement of the lifetime further verifies that SEDL could balance the electron and hole transport and promote the carrier injection into the active region. Furthermore, the average operation lifetime for sample D with ascending Al-content chirped SEDL is over 10,000 h at L50, which is adequate for the practical application.

## Conclusion

The effects of the chirped superlattice electron deceleration layer on the DUV LEDs are investigated experimentally and numerically. The results indicate that chirped SEDLs are able to equilibrate electron and hole injection into the active region, which promotes the radiative recombination in the first few quantum wells near n-type layers. The increase of radiative recombination further leads to the enhancement of DUV LED device performance. The AlGaN-based DUV LEDs have been fabricated into 762 × 762 μm^2^ chips, exhibiting single peak emission at 275 nm. External quantum efficiency of 3.43% and operating voltage of 6.4 V are measured at a forward current of 40 mA, demonstrating that the wall-plug efficiency is 2.41% of the DUV LEDs with ascending Al-content chirped SEDL. The lifetime of the DUV LED with ascending Al-content chirped SEDL is measured to be over 10,000 h at L50, due to the carrier injection promotion. Further improvement can be expected by introducing laser lift-off, surface roughening, reflecting electrode, and encapsulation. In general, the designed DUV LED with chirped SEDL shows satisfactory electrical property, favorable optical performance, and desirable reliability, which is promising for high-efficiency water purification and surface sterilization.

## Data Availability

All the data and materials in the manuscript are available.

## Abbreviations

APSYS: Advance Physical Model of Semiconductor Devices
BF-STEM: Bright-field scanning transmission electron microscopy
CIE: Carrier injection efficiency
DUV: Deep ultraviolet
EBL: Electron blocking layer
EQE: External quantum efficiency
FWHM: Full width at half maximum
HR-XRD: High-resolution X-ray diffraction
IQE: Internal quantum efficiency
LED: Light-emitting diode
LEE: Light extraction efficiency
LOP: Light output power
MOCVD: Metal-organic chemical vapor deposition
SEDL: Superlattice electron deceleration layer
SRH: Shockley-Read-Hall
TDD: Threading dislocation density
TMA: Trimethylaluminum
TMG: Trimethylgallium
WPE: Wall-plug efficiency
XRC: X-ray rocking curve
